# 0474. Circulating mitochondrial dna and vitamin d in critical illness

**DOI:** 10.1186/2197-425X-2-S1-O16

**Published:** 2014-09-26

**Authors:** AA Litonjua, LE Fredenburgh, RM Baron, L Gazourian, AF Massaro, K Nakahira, AMK Choi, KB Christopher

**Affiliations:** Brigham and Women's Hospital, Pulmonary and Critical Care Division, Boston, MA USA; Weill Cornell Medical Center, Department of Medicine, New York, NY USA; Brigham and Women's Hospital, Renal Division, Boston, MA USA

## Introduction

Mitochondrial dysfunction and an impaired autophagic response is associated with mortality in experimental sepsis. Vitamin D is shown to upregulate NOD2 which is linked to autophagy. Autophagy regulates innate immunity via inhibition of mitochondrial DNA (mtDNA) release via by the inflammasome. Circulating plasma mt-DNA is a robust predictor of mortality in ICU patients.

## Objectives

We hypothesized that critically ill patients with low plasma 25(OH)D would have high levels of circulating plasma mt-DNA.

## Methods

We performed a prospective observational cohort of MICU patients at the Brigham and Women's Hospital from 2008-2010 [n=49]. The exposure of interest was 25(OH)D categorized *a priori* as deficiency (25(OH)D ≤ 15 ng/mL) and measured via competitive chemiluminescence immunoassay. Circulating plasma mitochondrial DNA (mt-DNA) were assessed by measuring copy number of the NADH dehydrogenase 1 gene using quantitative real-time PCR. Plasma 25(OH)D was measured from the same plasma sample as mtDNA. Adjusted associations were estimated through fitting of multivariable logistic regression models including covariate terms for potential confounders of interest. Time-to-event analysis was performed using Cox proportional hazard regression.

## Results

Of the cohort patients studied, 37% were women and 84% were white. The mean age at critical care initiation was 53.5 years (SD 14.2). The mean APACHE II score was 24.5 (SD 8.3), while 100% of the cohort had SIRS, 71% had a source of infection identified and 18% had ARDS. The mean plasma 25(OH)D was 20.6 (SD 11.4) and 28.6% of the study cohort had plasma 25(OH)D ≤ 15 ng/mL. Gross unadjusted 30-day mortality was 26.5%. The mean (SD) plasma 25(OH)D was significantly higher in patients with mtDNA < 4000 copies/ml [25(OH)D 22.0 (11.2) ng/ml] relative to those with mtDNA ≥ 4000 copies/ml [25(OH)D 14.9 (10.9) ng/mL]; P < 0.001. When the cohort was analyzed with the exposure as plasma 25(OH)D and the outcome as plasma mtDNA ≥ 4000 copies/ml, we find that patients with plasma 25(OH)D < 15 ng/mL have a significantly higher odds of having plasma mtDNA ≥ 4000 copies/ml (unadjusted OR= 7.50, 95% CI 1.53-36.79; P=0.013). 25(OH)D in the cohort remains a significant predictor of plasma mtDNA ≥ 4000 copies/ml following adjustment for age, gender, race and APACHE II (adjusted OR= 13.19, 95% CI 1.69-103.34; P=0.014). Cox proportional hazard multivariable regression modeling, adjusting for *a priori* defined covariates comprising APACHE II score, age, sex, race, and plasma 25(OH)D, showed that mtDNA was predictive of all cause mortality following critical care (HR, 4.02; 95% CI, 1.14-14.20 ng/ml).

## Conclusions

25(OH)D levels are associated with circulating mtDNA levels. This study cannot determine a relationship between 25(OH)D and mtDNA beyond association but raises the question as to whether vitamin D-mediated pathways might play a role in linking autophagic and inflammasome processes.

